# Chiral Switch: Between Therapeutical Benefit and Marketing Strategy

**DOI:** 10.3390/ph15020240

**Published:** 2022-02-17

**Authors:** Gabriel Hancu, Adriana Modroiu

**Affiliations:** Department of Pharmaceutical and Therapeutic Chemistry, Faculty of Pharmacy, George Emil Palade University of Medicine, Pharmacy, Science and Technology of Târgu Mureș, 540142 Târgu Mureș, Romania; adriana.modroiu@umfst.ro

**Keywords:** chiral drugs, chiral switch, pure enantiomers, racemates

## Abstract

Chirality of pharmaceutical substances is an important aspect in drug research because it determines how enantiomers will interact with chiral biological targets. Enantiomers of a chiral drug can have different pharmacokinetic and pharmacological profiles; consequently, using a single pure enantiomer instead of a racemate can enhance the effectiveness and/or safety of the treatment. The tendencies of modern pharmaceutical industry regarding the current market of chiral drugs are divided between the chiral switch of previously used racemates and the development of new enantiopure drugs. The term chiral switch refers to the replacement on the market of a previously approved racemate with its single enantiomer version. The potential advantages of chiral switch can be related to a higher therapeutic index due to better potency, selectivity and fewer adverse effects, faster onset of action and exposure of the patient to lower drug dosages. However, chiral switch is also a strategy that permits manufacturers to keep market exclusivity for chiral pharmaceuticals that have lost their patent protection, even if the pure enantiomers have not demonstrated higher effectiveness or safety profile compared with the racemates.

## 1. Introduction

From the stereochemistry point of view, drugs can be divided into achiral, racemic and enantiopure. Approximately 50% of the small molecules used currently in therapy are chiral, containing at least one center of asymmetry in their structure, however their large majority are marketed as racemates and only about 25% in the form of pure enantiomers [1].

Living organisms are made up of enantiomerically pure chiral substances as in the living world all amino acids have an *L*-absolute configuration while all carbohydrates have a *D*-absolute configuration. As a result, essential physiological processes are stereoselective and are using just one of all potential enantiomers [2].

In the field of chiral pharmaceuticals, the turning point was the year 1992, when the Food and Drug Administration (FDA) issued a policy statement regarding the development and approval of chiral drugs; the policy adopts a rather strict approach toward chiral drugs, offering detailed guidelines on their assessment [3]. A similar policy was adopted later in 1994 by the European Medicines Agency (EMA). Both regulatory agencies have indicated rules that explicitly specify that developing an enantiopure medicine should be desired. Current regulations still leave the door open to produce racemates as long as there is evidence that the administration of the racemate will lead to therapeutic advantages in comparison with the single enantiomer [4].

Many chiral drugs are still used clinically as racemates, although it has been established that the pharmacokinetic and pharmacological profiles of the enantiomers differ. Thus, it is known and demonstrated that the desired pharmacological effect is generally limited to only one of the enantiomers, called eutomer, while the other enantiomer, called distomer, may be inactive, less active or in some cases may even be responsible for the adverse effects of the racemate [1,5].

In an achiral environment, enantiomers have identical physical and chemical properties, but in a chiral environment, enantiomers with different pharmacological and pharmacokinetic properties can act as different drugs [6]. Enantiomers can have different pharmacokinetic and pharmacological profiles because the human body is a chiral environment (being built of chiral structures: proteins, amino acids, enzymes, phospholipids), and the pharmacological activity of drugs depends mainly on their interaction with different chiral targets, such as proteins (receptors, enzymes), nucleic acids (DNA, RNA) or biomembranes (phospholipids, glycolipids) [7].

An enantiopure drug is a substance that comes in a single purified enantiomeric form. The use of pure enantiomers in therapy is still limited in some cases by the laborious process involving enantioselective synthesis methods, high prices of pure enantiomers and the difficulties in developing efficient enantioselective methods for their analysis [8]. However, in the last 25 years there has been an increasing trend towards the introduction in therapy of pure enantiomers, as demonstrated by the relatively large number of officinal enantiopure substances present in modern pharmacopoeias (European Pharmacopoeia 10th edition, United States Pharmacopoeia 44).

In the last two decades, in addition to the introduction of new pure enantiomers into therapy, several “old” racemates have been re-evaluated to be replaced with pure enantiomers [9]. Thus, by the end of the 1990s and the beginning of 2000s, the “chiral switch” process had become increasingly prominent, this term referring to the replacement of a chiral drug used in the form of a racemate with its eutomer; a change in the chirality status being the most important requirement for a chiral switch. The chiral switch phenomenon has led to the presence on the pharmaceutical market of drugs available at the same time both in the form of pure enantiomers and racemates [10].

Potential therapeutic benefits of the chiral switch strategy are related to an improved therapeutic index by increasing selectivity and potency towards receptors, reducing adverse effects, decreasing inter-individual variability of the therapeutic response, decreasing administered doses, improving pharmacokinetic profile, and decreasing drug–drug interactions [9,11,12].

However, this process is often linked to patent expiration of the racemate and has led to allegations of “evergreening” between original and generic manufacturers. The strategy is efficient as the pure enantiomer quickly absorbs the market share of the racemic precursor and redirects the use of generic versions of the racemate [9,13].

FDA and EMA cannot by law require active comparators in clinical trials for drug approval, consequently pure enantiomer products can enter the market by showing superior efficacy over placebo, rather than their precursor racemate [3,13].

Issues regarding chiral switch strategy were discussed in several previously published reviews by Tucker (2000) [11], Agranat et al. (2002) [10] and Gellad et al. (2011) [12]; however, those published in the period when chiral switch was a real “trend” do not entirely characterize the current “state of the art”.

The objective of the current review is a retrospective analysis on the tendencies of the current chiral drug market focusing on the most successful chiral switches in the past decades.

## 2. Chiral Switches in Therapy

The turning point in the regulation of chiral drugs was the thalidomide (2-(2,6-dioxopiperidin-3-yl)isoindole-1,3-dione) tragedy in the 1960s. Thalidomide, a glutamic acid derivative, was used as a racemate for its sedative-hypnotic effects but also for treating morning sickness in pregnant women. However, while *R*-thalidomide was responsible for the sedative-hypnotic effect, *S*-thalidomide exhibited teratogenic (mutagenic) effects, which led to the birth of thousands of children with birth defects (phocomelia) around Europe [14]. The theory that the tragedy could be avoided in this case by using a single enantiomer is misleading, because it was later demonstrated that the “safe” *R*-thalidomide suffers an in vivo chiral inversion to the “teratogenic” *S*-thalidomide. Even if the enantiomers of thalidomide have different toxicity, their quick in vivo racemization makes the potential chiral switch strategy inefficient [15].

The case of thalidomide demonstrates that a chiral molecule, as well as its multiple chiral and achiral metabolites, are responsible for a variety of pharmacological effects; consequently, determining the specific pharmacological action of each enantiomer is sometimes circumstantial and always challenging when a racemate is administered.

One of the best-known examples of chiral switch are the ones of aryl propionic acid derivatives (profens) anti-inflammatory drugs (ibuprofen, ketoprofen). These drugs were all introduced in therapy as racemates, except for naproxen, used in therapy as *S*-naproxen (*R*-naproxen is hepatotoxic). In the case of profens, *S*-enantiomers are mainly responsible for the anti-inflammatory effect related to the cyclooxygenase-2 (COX-2) inhibition. However, in vivo, there is a metabolic bioconversion of the distomer (*R*-enantiomer) into the eutomer (*S*-enantiomer). The configurational inversion process, together with the stereospecificity of action, presented pharmaceutical companies with a basis for using the *S*-enantiomers of this type of medication in therapy [16].

**Figure 1:**
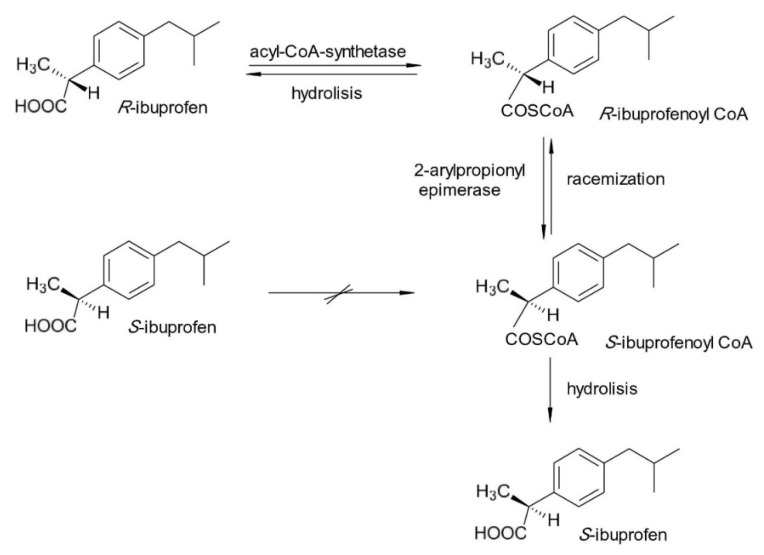
Chiral metabolic inversion of Ibuprofen.

In the case of ibuprofen (2-[4-(2-methylpropyl)phenyl]propanoic acid), the fact that *S*-ibuprofen (dexibuprofen) is an over 100-fold more potent COX-2 inhibitor than *R*-ibuprofen was the trigger for the chiral switch [17]. However, racemic ibuprofen undergoes quick and significant unidirectional chiral inversion (approximately 60%), resulting in mostly *S*-ibuprofen and minimal *R*-ibuprofen in circulation (Figure 1). As a result, racemic and *S*-ibuprofen can be considered “almost bioequivalent”, although *S*-ibuprofen has a faster onset of action and lower interindividual variability in configurational inversion [18]. In addition, there is evidence that *R*-ibuprofen may contribute to the therapeutic effect of the racemate not only through chiral inversion to *S*-ibuprofen, but also through inhibition of COX-2, which contributes to the debate over the advantages of chiral switching in this case. Even if dexibuprofen was introduced in therapy in 1994, this switch has not been exploited in many countries because of difficulties in securing patents for dexibuprofen [18].

In the case of ketoprofen (2-(3-benzoylphenyl)propanoic acid), the metabolic chiral inversion of *R*-ketoprofen into *S*-ketoprofen (dexketoprofen) is lower than for ibuprofen (less than 10%) [19]. For ketoprofen the chiral switch is more straightforward, dexketoprofen being 2–4 more potent than the racemate. Furthermore, dexketoprofen is formulated as a salt, dexketoprofen trometamol, which brings other advantages related to rapid absorption from the stomach, rapid onset of action at a lower dosage, and improved tolerability (reduced potential for gastric ulceration) [20].

Another important chiral switch example is the one from the proton pump (H+/K+-ATPase) inhibitor (PPI) class (omeprazole, lansoprazole). PPIs were introduced in therapy as racemates, their chirality being generated by the presence in their structure of a chiral sulfur atom in the methylsulfinyl group which binds the benzimidazole and pyridine heterocycles. PPIs are pro-drugs; the active forms (sulfone) of PPIs are achiral, therefore the enantiomeric form does not influence the pharmacodynamic action of these substances but may be of particular importance during interaction with metabolic enzymes [21,22].

**Figure 2:**
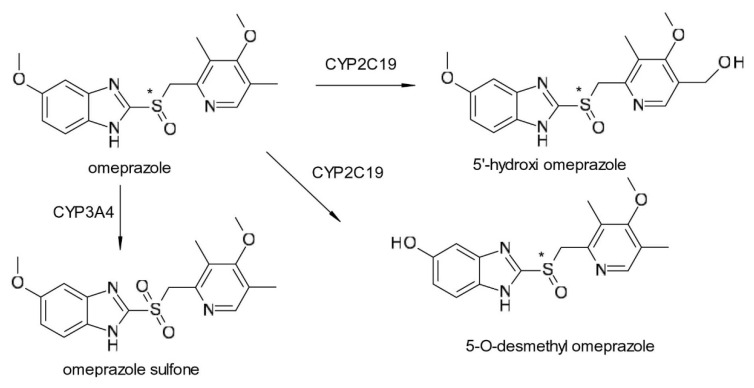
Metabolism scheme of omeprazole (* denotes the chiral center).

The two enantiomers of omeprazole (6-methoxy-2-[(4-methoxy-3,5-dimethylpyridin-2-yl)methylsulfinyl]-1H-benzimidazole) form the same main metabolites (hydroxy-omeprazole, desmethyl omeprazole and omeprazole sulfone), however their proportion may differ (Figure 2). In the case of *R*-omeprazole, hydroxylation by CYP2C19 is responsible for 98% of the liver clearance, while only 70% for *S*-omeprazole. The formation of the active sulfonyl derivative is also influenced by the CYP2A4 isoenzyme. This is especially important if we consider the genetic polymorphism of the CYP2C19 isoenzyme. The polymorphism of the microsomal isoenzyme CYP2C19 is based on a genetic mutation. Based on this autosomal recessive mutation, the population can be divided into two broad categories: fast metabolizers and slow metabolizers; slow metabolizers account for 3% of the Caucasian population and 15–20% of the Asian population. Esomeprazole clearance is less dependent on CYP2C19 than the racemate [23]. The difference between the hepatic metabolism of the two omeprazole enantiomers leads to certain therapeutic advantages of using esomeprazole over racemic omeprazole: higher bioavailability in fast metabolizers, and lower exposure in slow metabolizers (due to the alternative route of CYP2A4). The chiral switch of omeprazole to esomeprazole was developed on the premise that less interindividual variation (slow versus rapid metabolizers) and average higher plasma levels would provide higher dose efficiency [24].

In the case of lansoprazole (2-[[3-methyl-4-(2,2,2-trifluoroethoxy)pyridin-2-yl]methylsulfinyl]-1H-benzimidazole), it was observed that the *R*-enantiomer reaches a higher plasma level in both slow and fast metabolizers, and the lower level of *S*-lansoprazole (dexlansoprazole) appears to be offset by the more pronounced binding of plasma proteins to the *S*-enantiomer [25].

Although other racemic PPI medications (pantoprazole, rabeprazole) are structurally related to omeprazole, they lack the 5-methyl substituent at the pyridine ring and consequently are not susceptible to CYP2C19 5-methyl hydroxylation; therefore, their enantiomers are less likely to exhibit polymorphism interindividual metabolic variance [22].

**Figure 3:**
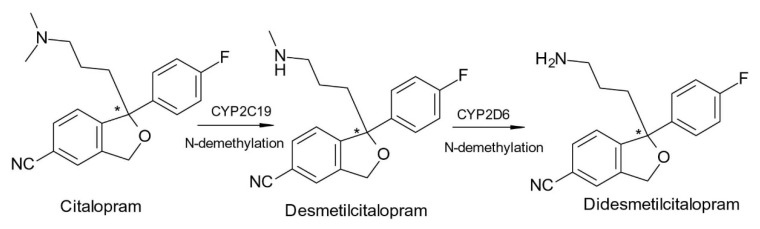
Metabolism scheme of Citalopram (* denotes the chiral center).

In the case of the selective serotonin reuptake inhibitor (SSRI) antidepressant, citalopram (1-[3-(dimethylamino)propyl]-1-(4-fluorophenyl)-3H-2-benzofuran-5-carbonitrile), the antagonism of serotonin reuptake is strongly related to the activity of *S*-citalopram (escitalopram), which is over 100-fold more potent than *R*-citalopram. However, *S*-citalopram plasma concentrations are roughly one-third of those of the total drug after racemic citalopram administration [26]. Citalopram is metabolized via demethylation to an active metabolite, desmethylcitalopram (Figure 3), which is roughly six times less effective than the parent drug in the case of the *S*-enantiomer, but four times more potent in the case of the *R*-enantiomer. Desmethylcitalopram is further N-demethylated to didesmethylcitalopram, which has limited SRI effect and is found in low amounts in plasma. *S*-citalopram administration has various advantages over racemic citalopram, including higher potency, lower dosages, and avoidance of *R*-citalopram-related side effects [27].

**Figure 4:**
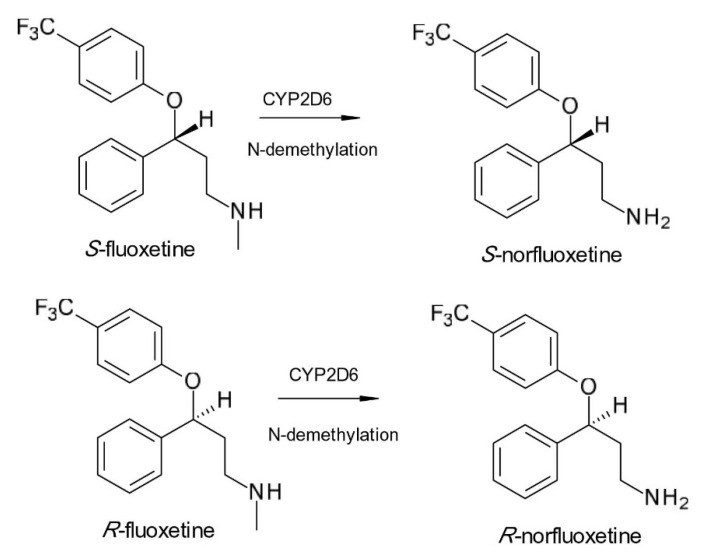
Stereoselective metabolism scheme of fluoxetine.

An unsuccessful attempt of chiral switch was made in the case of another SSRI, fluoxetine (N-methyl-3-phenyl-3-[4-(trifluoromethyl)phenoxy]propan-1-amine). Fluoxetine is converted stereoselectively by N-demethylation to an active chiral metabolite, norfluoxetine, which is likewise a powerful SSRI (Figure 4). *R*-fluoxetine and *S*-fluoxetine have similar SSRI potencies; however, in the case of active metabolites, *S*-norfluoxetine is a more powerful SSRI than *R*-norfluoxetine. Furthermore, plasma concentrations of *S*-norfluoxetine were shown to be higher than those of *R*-norfluoxetine in patients treated with racemic fluoxetine [28]. The use of *R*-fluoxetine was predicted to result in less variable fluoxetine and norfluoxetine plasma levels than in the case of racemic administration. Clinical trials were conducted to assess the safety and efficacy of *R*-fluoxetine; however, large doses of *R*-fluoxetine were shown to cause a minor but statistically significant lengthening of cardiac repolarization (QTc prolongation) in phase III clinical research, and the studies were halted. *S*-fluoxetine has also been studied in clinical trials for migraine prevention, but the results were not successful [29].

Albuterol (4-[2-(tert-butylamino)-1-hydroxyethyl]-2-(hydroxymethyl)phenol), also known under the name salbutamol, is a β2-receptor agonist used as a bronchodilator in asthma. Its bronchodilator effect resides mainly in *R*-albuterol (levalbuterol), which exhibits an over 60-fold higher potency than *S*-albuterol; while *S*-albuterol indirectly antagonizes the effects of *R*-albuterol and may have proinflammatory effects [30]. Furthermore, there are differences between the pharmacokinetic profiles of the enantiomers, as *S*-albuterol is cleared more slowly than the eutomer and tends to accumulate in the lungs, which can cause enhanced bronchial hyperresponsiveness. No significant chiral inversion of the enantiomers was identified after administration. The chiral switch of albuterol to levalbuterol was based on these pharmacokinetic and pharmacodynamic differences [31].

Formoterol (N-[2-hydroxy-5-[1-hydroxy-2-[1-(4-methoxyphenyl)propan-2-ylamino]ethyl]phenyl]formamide) is another β2-receptor agonist bronchodilator; it has 2 chiral centers in its structure, which generates the existence of 4 stereoisomers, and was used initially as a mixture of *R*,*R*- and *S*,*S*-stereoisomers [32]. As in the case of albuterol, there is a stereoselectivity in its action, with *R*,*R*-formoterol (arformoterol) having a 100-fold higher potency than *S*,*S*-formoterol towards β2 receptors [31].

Bupivacaine (1-butyl-N-(2,6-dimethylphenyl)piperidine-2-carboxamide) is a long-lasting local anesthetic, which is associated with cardiotoxicity. *S*-bupivacaine (levobupivacaine) proved to be less cardiotoxic than *R*-bupivacaine or racemic bupivacaine, while maintaining a similar anesthetic effect, with longer duration of action and less vasodilatation [33]. Many studies have compared levobupivacaine to bupivacaine, with the majority (but not all) demonstrating that levobupivacaine is less harmful [34]. Levobupivacaine has been found in several studies to have much fewer effects on cardiovascular function after i.v. administration than racemic bupivacaine [35]. The same properties apply to another local anesthetic, ropivacaine (propyl homologue of bupivacaine) [36].

Cetirizine (2-[2-[4-[(4-chlorophenyl)-phenylmethyl]piperazin-1-yl]ethoxy]acetic acid) is a H1 antihistaminic drug used as an antiallergic; its effect resides mainly in its *R*-enantiomer (levocetirizine), which is 10-fold more potent antihistaminic than the *S*-enantiomer. No significant racemization of levocetirizine was identified after administration [37].

Ketamine (2-(2-chlorophenyl)-2-(methylamino)cyclohexan-1-one) is a parenteral administered general anesthetic, which induces dissociative anesthesia. The use of *S*-ketamine (esketamine), which is more efficient as an analgesic and anesthetic through N-methyl-*D*-aspartate (NMDA) receptor antagonism, leads to shorter recovery after administration, increased tolerance and diminished side-effects (hallucinations and agitation). Currently both racemic ketamine and especially esketamine are used at lower sub-anesthetic doses and are considered promising options in the treatment of chronic pain and treatment-resistant depression [38].

Methylphenidate (methyl 2-phenyl-2-piperidin-2-ylacetate) is a stimulant drug used to treat attention deficit-hyperactivity disorder (ADHD) and narcolepsy; it has two chiral centers in its structure, which generates the formation of four stereoisomers (initially it was used as a mixture of *R*,*R*- and *S*,*S*-methylphenidate). *R*,*R*-methylphenidate (dexmethylphenidate) is approximately 10-fold more potent than *S*,*S*-methylphenidate in the inhibition of dopamine and norepinephrine. In addition, methylphenidate undergoes enantioselective metabolism, the absolute bioavailability being higher for the *R*,*R*-enantiomer [39].

Ofloxacin (7-fluoro-2-methyl-6-(4-methylpiperazin-1-yl)-10-oxo-4-oxa-1-azatricyclo[7.3.1.05,13]trideca-5(13),6,8,11-tetraene-11-carboxylic acid) is a 2nd generation fluoroquinolone antibacterial with a broad spectrum of action against Gram-positive and Gram-negative bacteria. *S*-ofloxacin (levofloxacin) binds more effectively to the DNA gyrase enzyme and to topoisomerase IV than *R*-ofloxacin; *S*-ofloxacin has a 2-fold more potent antibacterial activity over the racemate, while *R*-ofloxacin is pharmacologically inactive [40,41].

Fenfluramine (N-ethyl-1-[3-(trifluoromethyl)phenyl]propan-2-amine) is a sympathomimetic stimulant with appetite suppressing properties deriving from amphetamine used in the short-term treatment of obesity (combination with phentermine); its anorectic effect being linked mainly to *S*-fenfluramine (dexfenfluramine) enantiomer. Both the racemic mixture and its pure enantiomer dexfenfluramine were used in therapy; as it was hoped that the use of the single enantiomer will increase potency and tolerance, but they were both withdrawn in 1997 due to cardiovascular side effects; being associated with valvular heart lesions and pulmonary hypertension [42].

Two failed attempts of chiral switch were made in the β-blockers class, a class in which every substance is chiral. Differences between the pharmacokinetic and pharmacodynamic profiles of the enantiomers were identified, but only timolol is used in therapy in the form of a pure enantiomer (*S*-timolol) [43].

**Figure 5:**
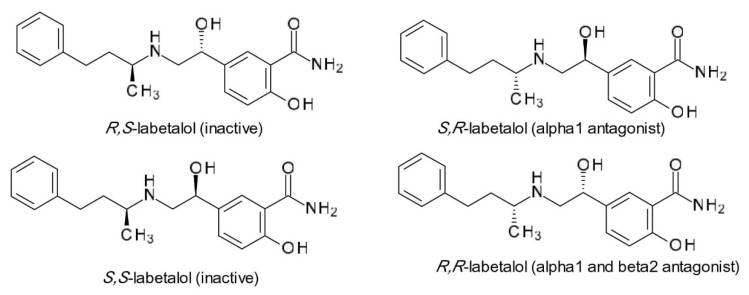
Chemical structures of labetalol stereoisomers.

Labetalol (2-hydroxy-5-[1-hydroxy-2-(4-phenylbutan-2-ylamino)ethyl]benzamide) a non-selective β-adrenergic blocker with associated α_1_-adrenergic blocker effect, has two chiral centers in its structure, which generates the existence of four stereoisomers. Two of them, *S*,*S*- and *R*,*S*-labetalol are inactive, *S*,*R*-labetalol is a α1 antagonist, while *R*,*R*-labetalol has both α1 and β2 antagonist effects (Figure 5) [44]. A chiral switch was attempted for dilevalol, the *R*,*R*-stereoisomer of labetalol, which, although it had the benefit of not being associated with orthostatic hypotension, was never commercialized due to severe hepatotoxicity, not reported when racemic labetalol was administered [45].

Sotalol (N-[4-[1-hydroxy-2-(propan-2-ylamino)ethyl]phenyl]methanesulfonamide) is a nonselective β-adrenergic blocker used as a class III antiarrhythmic, with a chiral carbon atom in its structure. *R*-sotalol has both a β-blocker and a potassium channel blocker effect, while *S*-sotalol has potassium channel blocking activity, its affinity towards β receptors being low. The results of the SWORD (Survival With ORal *D*-sotalol) study showed that administration of optically pure *S*-sotalol increased mortality (fatal arrhythmias) in patients with myocardial infarction compared with placebo [46,47].

Examples of racemate drugs that have been switched successfully to the single-enantiomer version are presented in Table 1.

## 3. Discussion

Taking into consideration the current FDA and EMA regulations, the current tendency of the pharmaceutical industry favors the development of new enantiomerically pure compounds to the detriment of the chiral switch practice to single enantiomers from already registered racemates [48]. A review regarding the current market of chiral drugs, comparing chiral switches to introduction of new enantiomeric pure drugs, was published in 2018 by Calcaterra and D’Acquarica; the review concludes that although the chiral switch strategy has been a prominent strategy of pharmacological development, notably between 1994 and 2011, it was less frequently used in the last decade [49].

**Figure 6:**
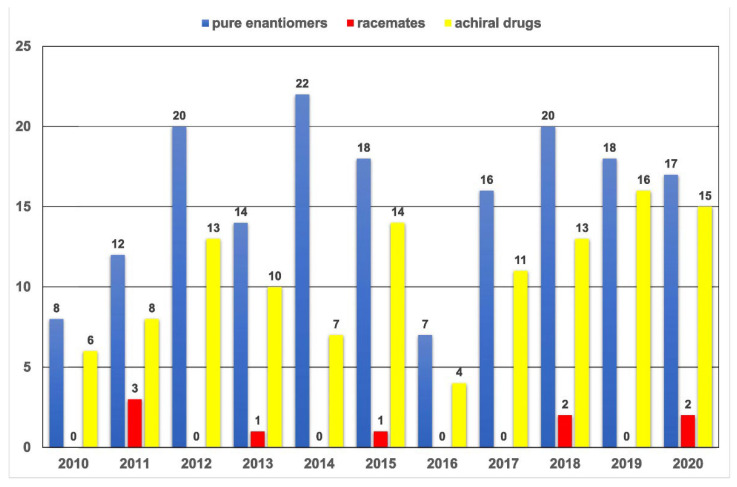
Comparison on the number of yearly FDA approved drugs over the period 2010–2020 (pure enantiomers vs. racemates vs. achiral drugs).

Figure 6 shows the number of yearly approved drugs by the FDA according to three selected categories (single enantiomers, racemates and achiral drugs) in the period 2010–2020. It is noticeable that in the last 10 years, the FDA approved less than 10 racemates, and the large majority of the approved substances were in the form of pure enantiomers [50].

The single enantiomer introduced in therapy as a result of chiral switch has a similar profile and indications as the “parent” racemate but can present several therapeutic advantages: more predictable pharmacodynamic profile, improved therapeutic index and safety, reduced possibility for drug–drug interactions, faster onset of action and patient exposure to lower dosages [8,12,51].

Patients have benefited from a single-enantiomer chiral switch in several circumstances, especially when the pharmacological action is concentrated in one of the two enantiomers (citalopram—escitalopram, ofloxacin—levofloxacin), or when the single enantiomer is less toxic than its racemate (bupivacaine—levobupivacaine).

However, there have been cases in which single-enantiomer medications generated from blockbuster racemates had minimal clinical benefit over the racemate (ibuprofen, PPIs), and their release onto the market was likely used by pharmaceutical corporations as a patent-protection tactic against generic competition.

Another interesting example is the one of fenfluramine, which was switched successfully to dexfenfluramine but later withdrawn from therapy due to an unfavorable safety profile.

Not all of the attempted switches have been successful, and sometimes unanticipated adverse effects were reported, and the chiral switch process was stopped (fluoxetine, labetalol, sotalol).

Table 2 presents therapeutical advantages of using pure enantiomers in several successful chiral switch processes.

Regardless of why a single-enantiomer medicine is being developed, the FDA and EMA review and approval process remains identical; as regulatory agencies normally approve new pharmaceuticals based on their efficacy in achieving a specific goal, and an improvement in action over placebo is commonly recognized. To obtain FDA or EMA approval, manufacturers of single-enantiomer medications are not obliged to undertake randomized clinical studies to compare their products to racemates. An interesting investigation was published in 2021 by Long et al. in which randomized clinical trials directly comparing single-enantiomer drugs to a previously used racemic precursors for efficacy or safety differences were evaluated. Fifteen drugs subject to a chiral switch were evaluated, and for nine of them, no randomized clinical trials that showed enhanced effectiveness or safety when compared to their racemic predecessors were found [52]. It is interesting that more than half of these randomized clinical trials involved bupivacaine versus levobupivacaine. According to the findings of this systematic analysis, newly approved single-enantiomer medications are seldom directly compared to racemic precursors, and when they are, rarely have they been shown to deliver enhanced effectiveness or safety [52]. It should be acknowledged that the regulatory authorities do not have the legal authority to demand comparative effectiveness testing of single enantiomers to the previously registered racemates, prior to approval [9,49,52].

However, there have been situations when the innovator company neglected to patent the single enantiomers when the medications were first developed, and this allowed other companies to produce the single isomer and, as a result, engage into license arrangements with the racemate’s inventors. However, usually, enantiomer patents are issued by the innovator company with priority dates that are much later than those of the equivalent racemic patents [13]. It is critical for the patent owner of a blockbuster racemic drug to launch the single-enantiomer drug before the racemic drug’s patents expire and generic copies of the drug enter the market. The majority of chiral switch medications’ enantiomer patents have been challenged. Lack of originality, lack of value, inadequacy of disclosure, misleading suggestion, misrepresentation, and double patenting are all common reasons for generic companies to contest the validity of enantiomer patents [9,53].

There are several examples of well-timed switches of racemic pharmaceuticals (profens, PPIs) with patents due to expire, in which quick and cost-effective procedures have been designed to allow efficient development and regulatory licensing of switched single enantiomer pure medications [9,49].

The launch of a single-enantiomer medicine is frequently timed to coincide with the launch of generic competition for its racemic counterpart, as many single-enantiomer medications have become commercial blockbusters, displacing generic versions of racemic predecessors from the market [54].

## 4. Conclusions

The increased development of individual isomers at the cost of racemates has been aided by this understanding of the implication of stereochemistry in the pharmacological effect of a chiral drug, combined with developments in chemical technology and further forced by regulatory requirements. In modern chiral drug development, there are two main variants: development of an enantiomerically pure drug or switching from a racemic molecule to its eutomer in a pure form (chiral switch).

Chiral switch is a controversial practice, as in some cases the use of the pure enantiomer does not provide enough clinical evidence for its benefit and is used by pharmaceutical companies to maintain sales as the initial racemate reaches the end of its patent on the market. Despite the increasing tendency of switching racemates to pure enantiomers in therapy, most of these enantiomeric pure drugs approved by the FDA and EMA were not specifically compared with their racemic counterpart, and there is not always indication of positive changes in treatment outcome.

When the pharmacological effects of the enantiomers of a chiral drug differ sufficiently from each other and from the racemate, it is feasible to get a patent for one or both enantiomers in addition to the racemate’s patent. Several blockbuster medications were synthesized and registered initially as racemates, and their replacement with single isomers might be considered as an excellent approach of prolonging patent franchise and safeguarding against generic competition.

Nonetheless, in many situations, the use of the pure enantiomer instead of the racemate may be in the benefit of patients, especially in the cases when the distomer is responsible for undesirable effects, when manufactures may seek single enantiomer production to improve safety and effectiveness.

In the current review, we have presented how the chiral switch’s basic notion has evolved over the last 25 years. The original hypothesis on which chiral switch strategies were built, that existing racemates would offer a plentiful supply of new single enantiomer drugs, has proven difficult to realize; however, in some individual cases, the chiral switch presented a valuable alternative for existing racemate owners to obtain line extensions, particularly if the switch was marketed before the racemate’s patent expires.

The goal of development of novel therapeutic alternatives is to improve efficacy and/or safety; the choice between homochiral drugs and racemates should be based on therapeutic benefits, potential undesirable side effects, and development costs.

## Figures and Tables

**Table 1:** Racemates that were “chiral switched” in therapy to pure enantiomers (* denotes the chiral centers).

No.	Racemate	Active Enantiomer	Chemical Structure	Pharmacological Activity
1	*R*,*S*-Albuterol	*R*-(−)-Albuterol (levalbuterol)	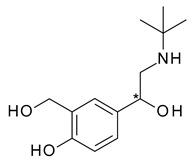	β_2_ adrenergic receptor agonist antiasthmatic
2	*R*,*S*-Bupivacaine	*S*-(−)-Bupivacaine (levobupivacaine)	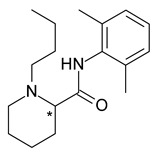	Local anesthetic
3	*R*,*S*-Cetirizine	*R*-(−)-Cetirizine (levocetirizine)	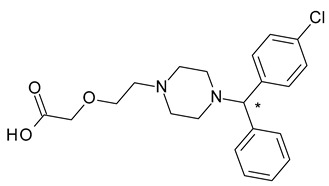	H1 antihistaminic antiallergic
4	*R*,*S*-Citalopram	*S-*(+)-Citalopram (escitalopram)	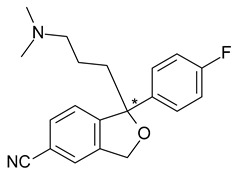	Selective serotonin reuptake inhibitor (SSRI) antidepressant
5	*R*,*S*-Fenfluramine	*S*-(+)-Fenfluramine (dexfenfluramine)	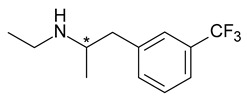	Anorectic—withdrawn from the market due to cardiovascular effects
6	*R*,*R*,*S*,*S*-Formoterol	*R*,*R*-(−) Formoterol (arformoterol)	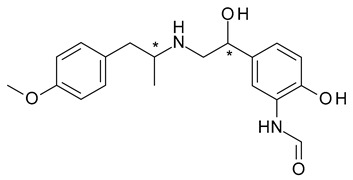	β2 adrenergic receptor agonist antiasthmatic
7	*R*,*S*-Ibuprofen	*S*-(+)-Ibuprofen (dexibuprofen)	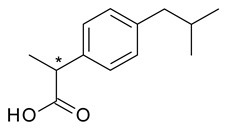	Nonsteroidal anti-inflammatory
8	*R*,*S*-Ketamine	*S*-(+)-Ketamine (esketamine)	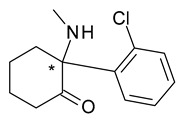	General anaesthetic
9	*R*,*S*-Ketoprofen	*S*-(+)-Ketoprofen (dexketoprofen)	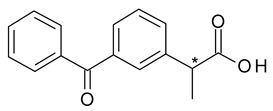	Nonsteroidal anti-inflammatory
10	*R*,*S*-Lansoprazole	*R*-(+)-Lansoprazole (dexlansoprazole)	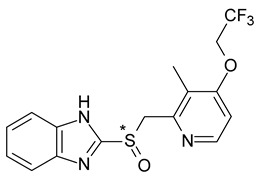	Proton pump inhibitor (PPI) antacid
11	*R*,*S*-Leucovorin (Folinic acid)	*S*-(−)-Leucovorin (levoleucovorin)	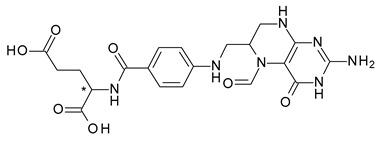	Folate deficiency, decreases the toxic effects of methotrexate and pyrimethamine treatments
12	*R*,*R*,*S*,*S*-Methylphenidate	*R*,*R*-(+)-Methylphenidate (dexmethylphenidate)	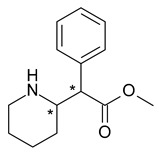	Stimulant in ADHD and narcolepsy
13	*R*,*S*-Milnacipran	*S*-(−)-Milnacipran (levomilnacipran)	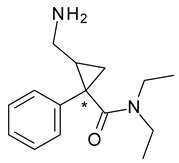	Serotonin and norepinephrine reuptake inhibitor (SNRI) antidepressant
14	*R*,*S*-Modafinil	*R*-(−)-Modafinil (armodafinil)	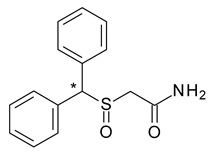	Eugeroic (wakefulness-promoter) in narcolepsy
15	*R*,*S*-Ofloxacin	*S*-(−)-Ofloxacin (levofloxacin)	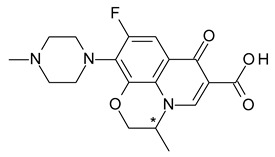	Fluoroquinolone antibacterial
16	*R*,*S*-Omeprazole	*S*-(+)-Omeprazole (esomeprazole)	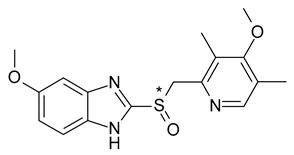	Proton pump inhibitor (PPI) antacid
17	*R*,*S*-Zopiclone	*S*-(+)-Zopiclone (eszopiclone)	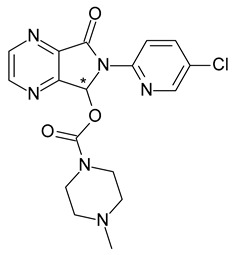	Sedative-hypnotic

**Table 2:** Examples of potential advantages of chiral switch.

No.	Racemate	Active Enantiomer	Potential Therapeutic Advantage
1	Albuterol	Levalbuterol	Increased potency, decreased development of airway hyperreactivity
2	Bupivacaine	Levobupivacaine	Decreased risk of cardiotoxicity
3	Cetirizine	Levocetirizine	Increased potency
4	Citalopram	Escitalopram	Increased potency, faster onset of action, improved tolerability profile
5	Formoterol	Arformoterol	Increased potency, decreased development of airway hyperreactivity
6	Ibuprofen	Dexibuprofen	Faster onset of action
7	Ketamine	Esketamine	Increased tolerance, shorter recovery time, decrease incidence of side-effects
8	Ketoprofen	Dexketoprofen	Increased potency, faster onset of action, decrease incidence of gastrointestinal side-effects (trometamol salt)
9	Lansoprazole	Dexlansoprazole	Less variable pharmacokinetic profile in some patients
10	Methylphenidate	Dexmethylphenidate	Increased potency
11	Ofloxacin	Levofloxacin	Increased potency
12	Omeprazole	Esomeprazole	Less variable pharmacokinetic profile in some patients

## Data Availability

Not applicable.
